# Analysis of Anthropometric and Body Composition Profile in Male and Female Traditional Rowers

**DOI:** 10.3390/ijerph18157826

**Published:** 2021-07-23

**Authors:** Alfonso Penichet-Tomas, Basilio Pueo, Sergio Selles-Perez, Jose M. Jimenez-Olmedo

**Affiliations:** Physical Education and Sports, Faculty of Education, University of Alicante, 03690 Alicante, Spain; alfonso.penichet@ua.es (A.P.-T.); sergio.selles@ua.es (S.S.-P.); j.olmedo@ua.es (J.M.J.-O.)

**Keywords:** rowing, anthropometry, somatotype, performance, talent identification

## Abstract

The anthropometric profile has a fundamental role in rowing performance and young talent detection. The objective of this study was to analyze the anthropometric profile, body composition, and somatotype in traditional rowers, and to analyze which variables can be used as predictors of rowing performance. Twenty-four rowers competing at national level participated in this study, thirteen men and eleven women. Significant differences (*p* < 0.001) were observed in the height of male rowers (large effect size, *d* = 1.8) and in body mass (very large effect size, *d* = 2.4). Also, muscle mass reached a higher percentage in male rowers (*d* = 3.7), whereas the sum of seven skinfolds (*d* = 2.0) and body fat percentage (*d* = 2.0) reached higher values in female rowers, all their difference being significant (*p* < 0.001) with very large effect size. The somatotype of male rowers was ecto-mesomorph (1.8-4.5-3.0), and the somatotype of female rowers was in the balanced mesomorph (2.8-3.8-2.6). A very strong correlation between height (*r* = 0.75; *p* = 0.002) and rowing performance was found in male rowers. Body mass (*r* = 0.70; *p* = 0.009) and muscle mass (*r* = 0.83; *p* = 0.001) showed also very strong correlation in female rowers. Finally, height was the best predictor of performance for male rowers (R^2^ = 0.56, *p* < 0.003) and muscle mass for female rowers (R^2^ = 0.68, *p* < 0.002). The anthropometric profile of male and female traditional rowers showed differences to be considered in training programs and talent selection.

## 1. Introduction

Rowing is a sport that consists of propelling a boat through the water using one or more oars. The difference with other sports that also use oars is that the oars are fixed to the body of the boat with the rower positioned towards the bow of the boat resulting in the production of different dynamic force components [1,2]. The main classification of rowing modalities differentiates between boats with a mobile seat or a fixed seat [3].

The modality with mobile seat boats is generally called Olympic rowing because only this modality includes Olympic boats. The seat of each rower is placed on rails that allow forward and backward movement. The legs produce almost half the power of the drive (46%) while the trunk around 32% and the arms 22% [4]. The competitions, which can last between 5 and 8 min depending on the type of boat and the category, are generally over the distance of 2000 m in calm waters [5]. On the other hand, in fixed seat boats, the seats do not move in the boat and the technical execution is different since the rowers are supported in the coccyx area. This technical difference that prevents the rower from using the legs in such a wide range of motion implies that the amplitude of the trunk degree is greater than in Olympic rowing [6]. This modality is also called traditional rowing because it is how rowing was originally practiced: Llaüt, with eight rowers and a coxswain [7], and Trainera, with 13 rowers and a coxswain [8]. In addition, traditional rowing courses are not held in parallel lanes, but between two and four lengths with one or three complete tacks, both in calm water and the sea. These technical and competitive differences between modalities, boats, and types of competition result in different functional and physiological demands [9], where anthropometric characteristics and body composition have a fundamental role in Olympic [10,11] and traditional [7,12] rowing performance.

Most studies about anthropometry, body composition, and somatotype have focused on Olympic rowing [13,14,15,16,17]. Furthermore, some studies have not only compared the different profiles based on weight or age category. The differences between male and female rowers have also been analyzed, finding differences and similarities in anthropometric characteristics that could determine not only training programs but also offering indicators to be able to perform talent detection programs [18,19,20]. Even De Larochelambert [10] determined which morphologies (tall and thin, tall and robust, small and thin, or small and robust) had a significant effect on speed for both male and female rowers. On the other hand, the research also seem to determine that there are anthropometric characteristics that are related to the level of rowing performance such as height and length measurements [21]. Taller rowers can perform a wider stroke in the water, and a greater stroke range is directly related to increased rowing performance [22]. A similar trend is found with the body mass of the rowers since higher values seem to be correlated with success in competition [14]. Higher body mass can be a disadvantage for performance in other sports where the athlete must shift their own weight. In rowing, the rower is sitting in the boat and his own weight does not seem to have a negative effect on performance. These characteristics are above all in the heavyweight categories because in the lightweight categories the differences and correlations with success in rowing are lower [20]. The studies carried out show that in the heavyweight categories the body mass does not have a negative impact, even a greater weight positively favors power production. However, in the lightweight categories this fact has not been demonstrated as strongly. The profitability of the rower may have a greater impact. Nevertheless, a higher percentage of body fat can be a disadvantage [18]. The body composition of rowers is characterized by a low percentage of fat mass and a mesomorph body type associated with a high development of muscle mass as somatotype [15,16]. It has been widely reported that anthropometric variables and success in rowing are associated, which shows that these characteristics could be used as predictors of performance [23]. Even carrying out a complete body composition study with quantitative and qualitative parameters can be used to plan specific training cycles in different periods of the season [24].

Research in traditional rowing about anthropometry and body composition profile is very limited. Some researchers have studied the relationship of anthropometric characteristics with traditional rowing performance and some of these findings seem to coincide with the Olympic rowing modality, such as a greater body mass and fat-free mass seem to have a positive impact on rowing performance [12]. However, there are some differences such as less muscle mass [25] or lower average height that seem not as important to performance in traditional rowers [8]. Traditional rowing boats require rowers of different heights and weights for hydrodynamic reasons to balance the boat in rough seas [8]. For example, Sebastia-Amat et al. [26] found that only body mass for male rowers and body muscle for female rowers were good predictors of performance in traditional rowing.

Further investigation of these differences between modalities and gender is essential to determine a complete profile of the traditional rower and for following objective criteria in talent recruitment programs. Furthermore, changes in some characteristics of body composition in rowers can be a performance advantage, so control and monitoring of body composition can be crucial for their success in competition [24]. For this reason and because there is also no scientific evidence of comparative studies that carry out a complete study of body composition profile of traditional rowers, the objective of this study is to analyze and compare the anthropometric profile, body composition, and somatotype in male and female traditional rowers. In addition, the present study also aims to analyze the anthropometric variables that influence rowing performance and which of them can be used as predictors of performance. Despite general variations between genders are expected, the differences will allow to create a differentiated profile of rowers competing at the national level and to verify that characteristics such as height and weight, among others, have a relevant role in rowing performance.

## 2. Materials and Methods

### 2.1. Participants

Twenty-four rowers competing at national level participated in this study, thirteen males (age: 27.3 ± 5.1 years; height: 182.1 ± 6.6 cm, body mass: 75.3 ± 5.3 kg) and eleven females (age: 27.7 ± 4.3 years; height: 169.9 ± 6.7 cm, body mass: 61.9 ± 6.0 kg). The requirement to participate was to have qualified for the national championship, with an experience of at least 3 years, and to regularly train a minimum of six days per week for 2–3 h/day, supervised by one of the authors who perform the physical preparation and monitoring of the athletes who have participated in the study. They were asked to refrain from eating for at least four hours before the measurements, not exercise on the day of the measurement [16] and not high intensity exercise the day before. The hydration guidelines were the same as those carried out for training, no specific hydration guidelines were given. All measurements were made at the same time of the day. Rowers who did not meet the selection criteria were excluded from the study. The Ethics Committee of the University of Alicante gave institutional approval for this study, in accordance with the Declaration of Helsinki (IRB UA-2020-07-21). The subjects were informed about the study and gave their written informed consent.

### 2.2. Procedure

The anthropometric assessment followed the guidelines set by the International Society for the Advancement of Kinanthropometry (ISAK) [27]. The measurements were performed by the same researcher with ISAK certification level II under fasting conditions at room temperature (22 ± 1 °C) [28,29]. All variables were measured on the right side of the body in duplicate and the mean value was recorded. Intra-observer technical error of the measurement (TEM), 5% for skinfolds and 1% for girths and breadths, was considered for the measurements.

Body mass and height were measured using a scale (model 707, Seca, Hamburg, Germany) to the nearest 0.1 kg and a stadiometer (Harpenden, Burgess Hill, UK) to the nearest 0.1 cm. Rowers were weighted and measured wearing only underwear with bare feet. Height was measured with the rower completely upright and the chin parallel with the ground. Body mass index (BMI) was computed as body mass (kg) divided by height squared (m^2^). Eight skinfolds (triceps, biceps, subscapular, iliac crest, supraspinal, abdominal, front thigh, and calf) were measured with a Holtain skinfold caliper to the nearest 0.2 mm and six girths (relaxed arm, tensed arm, thigh, medial calf, waist, and hip) were obtained using a Holtain bone breadth caliper to the nearest 0.1 cm (Holtain Ltd., Crymych, UK). The sum of eight skinfolds was examined following validated procedures [30]. Finally, three breadths (humerus, femur, and stylion) were measured with an anthropometric tape (Seca) to the nearest 0.1 cm. Fat, muscle, bone, and residual masses were calculated, as well as somatotype. To calculate the percentage of body fat, the formula of Withers et al. was used [30]. Muscle mass was determined using the methods of Lee et al. [31] and bone mass was calculated following the Rocha model [32]. The anthropometric somatotype was determined using the Carter and Heath equation [33], making a graphic representation of the results in a somatochart.

Once the anthropometric study was completed, the rowers performed an all-out 2000 m test on a rowing ergometer (Model D; Concept 2, Inc., Morrisville, VT, USA) with a coupling adapted for the reproduction of the traditional rowing stroke fixing the seat [25,34] and with a PM5 performance monitor to collect mean power output reached in the test, and its equivalence in time. All the rowers were familiar with the rowing machine and with the drag factor used: 160 for males and 140 for females. The rowers performed a 10-min warm-up before the test at moderate intensity between 70 to 80% of maximum heart rate (above 140 beats per min) at a stroke rate of 18–20 strokes per minute [26]. Power output, stroke rate and time to complete 2000 m rowing ergometer performance test were recorded.

### 2.3. Statistical Analysis

Descriptive analysis was presented by the mean, standard deviation (SD), minimum (min), and maximum (max) for all variables. Shapiro–Wilk statistical test was used to verify that the variables followed the normality criterion. Student’s *t*-test was used to compare anthropometric data between male and female rowers. Cohen’s *d* was used as a measure of the effect size of differences between male and female rowers and interpreted according to modified thresholds [35] for sports sciences [36] as trivial (<0.2), small (0.21–0.6), moderate (0.61–1.2), large (1.2–1.99), and very large (>2.0). Somatotype Attitudinal Mean (SAM) and Somatotype Attitudinal Variance (SAV) were calculated to describe the magnitude of the dispersion of somatotypes in both groups. Somatotype Attitudinal Distance (SAD), the distance in three dimensions between male and female groups, was used to compare somatotype group means. Pearson correlation coefficient (*r*) was used to determine relationships between each anthropometric variable with rowing performance. Effect sizes of relationships were assessed by Pearson’s correlations and coefficients of determination: trivial (<0.1), small (0.1–0.29), moderate (0.3–0.49), strong (0.5–0.69), very strong (0.7–0.89), nearly perfect (0.9–0.99), and perfect (1.0) [36]. A stepwise multiple regression analysis (R^2^ > 0.5) was used to analyze which anthropometric variables could be used to predict rowing performances. Statistical significance was set at *p* < 0.05. Statistical analyses were performed using Statistical Package for Social Sciences (SPSS v.26 for Windows, SPSS Inc., Chicago, IL, USA).

## 3. Results

Body mass, height, and BMI mean values were significantly higher (*p* < 0.05) in male rowers (182.1 ± 6.6 cm, 75.3 ± 5.3 kg, and 22.8 ± 1.3 kg/m^2^) than female rowers (169.9 ± 6.7 cm, body mass: 61.9 ± 6.0 kg 21.4 ± 1.0 kg/m^2^) with large to very large effect size, as shown in Table 1. However, the skinfolds of triceps, biceps, iliac crest, front thigh, and calf were significantly higher (*p* < 0.05) in female rowers than in male rowers, with moderate to very large effect size. Therefore, the mean of the sum of skinfolds also showed a larger value in female rowers (88.0 ± 17.6 mm) than in male rowers (58.5 ± 12.4 mm). This difference was statistically significant (*p* < 0.001) with very large effect size (*d* = 2.0). In contrast, most of the girths were significantly higher (*p* < 0.05) in male rowers than in female rowers, with moderate effect size on thigh girth (*d* = 0.9) and very large effect size on relaxed arm (*d* = 2.5), tensed arm (*d* = 3.4) and waist girths (*d* = 2.7). Finally, humerus (*d* = 2.7), femur (*d* = 1.8) and stylion breadths (*d* = 3.0) also reached statistically higher values in male rowers, with large to very large effect size.

Table 2 shows body composition and somatotype profile of male and female rowers which highlights that male rower reached larger values of muscle mass (46.7 ± 2.0%) than female rowers (39.1 ± 2.1%), with significant difference (*p* < 0.001; *d* = 3.7) and very large effect size. However, female rowers achieved higher fat (15.4 ± 3.1%) and residual masses (29.4 ± 1.9%) than male rowers (10.3 ± 2.1% and 26.4 ± 1.9%, respectively). This contrast showed significant differences (*p* < 0.001) and very large (*d* = 2.0) and large (*d* = 1.6) effect size, respectively.

**Figure 1:**
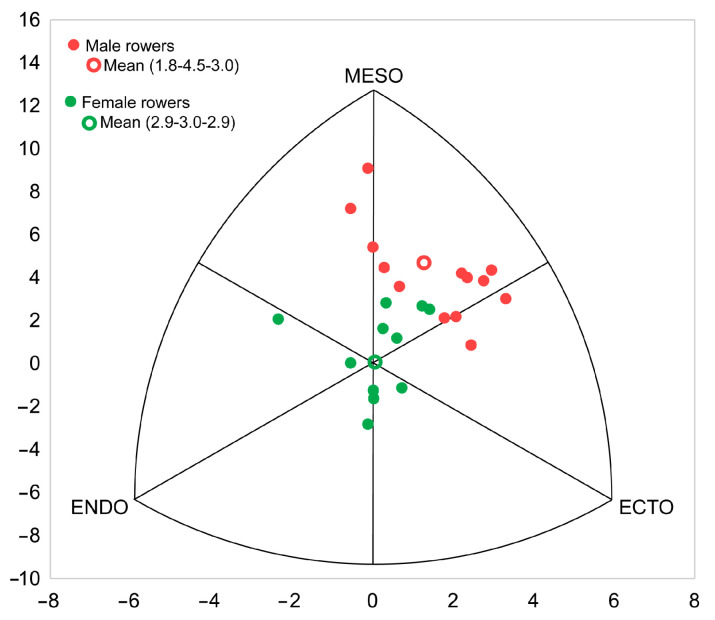
Somatochart of male and female rowers and mean somatotypes.

The comparative analysis of the somatotype between male and female rowers indicates that there are significant differences in endomorphy (*p* < 0.001; *d* = 2.0), with very large effect size, and mesomorphy (*p* < 0.001; *d* = 1.8), with large effect size. The mean somatotype of male rowers was mesomorph-ectomorph (1.8-4.5-3.8) and the mean somatotype of female rowers was balanced mesomorph (2.9-3.0-2.9) (Figure 1). Finally, SAM values were 1.1 in male rowers and 0.9 in female rowers where no significant differences between them, and the effect size was small (*d* = 0.2). The difference in SAD between male and female rowers was 1.0.

**Figure 2:**
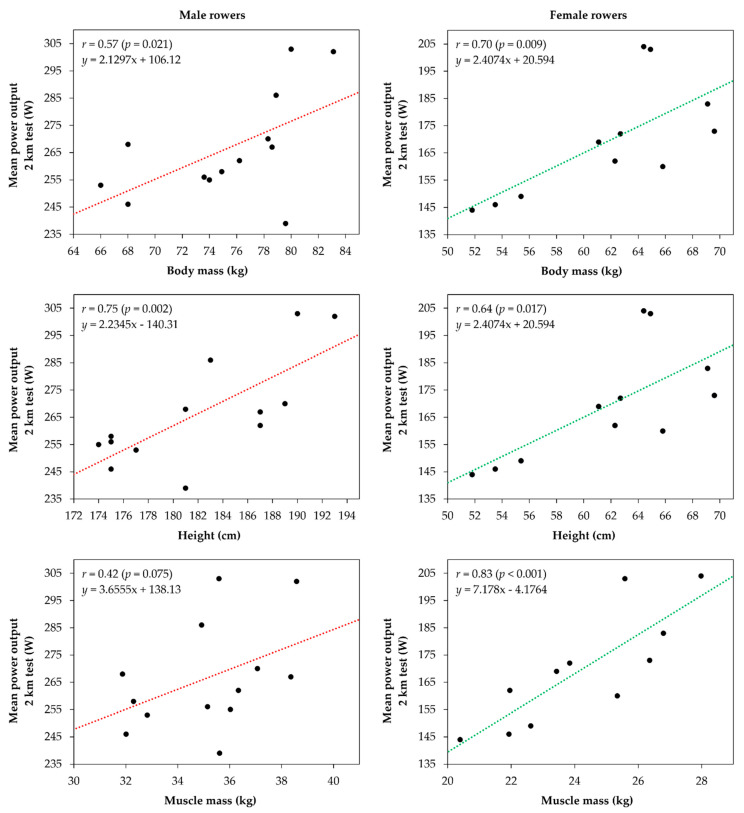
Relationships between anthropometric characteristics and rowing performance in male and female rowers.

Figure 2 shows the associations between anthropometric variables and rowing performance expressed in mean power output reached in 2000 m rowing test. The results show a strong correlation with body mass in male rowers (*r* = 0.57; *p* = 0.021) and a very strong correlation in female rowers (*r* = 0.70; *p* = 0.009). However, height was strongly correlated in female rowers (*r* = 0.64; *p* = 0.017) and very strongly correlated in male rowers (*r* = 0.75; *p* = 0.002) with performance. Finally, a very strong correlation was found between rowing performance and muscle mass in female rowers (*r* = 0.83; *p* = 0.001), while in male rowers the correlation was small (*r* = 0.42; *p* = 0.075).

Table 3 shows the results of the stepwise multiple regression analysis in male and female rowers by which height is the only predictor of rowing performance in male rowers, explaining 56% of variance (R^2^ = 0.56, *p* < 0.003). The single predictor of rowing performance in female rowers was muscle mass, explaining explained 68% of variance (R^2^ = 0.68, *p* < 0.002). The rest of anthropometric measures did not contribute significatively and were excluded from the prediction equation.

## 4. Discussion

The aim of this study was to analyze and compare the anthropometric profile, body composition, and somatotype in male and female traditional rowers. In addition, the present study aimed to analyze which variables can be used as predictors of rowing performance. As it is the first study that compares the anthropometric profile of traditional rowing between male and female rowers to determine reliable reference values, the selection criteria of the participants were to have classified for the national championship, to have an experience of at least 3 years and to regularly train a minimum of six days per week for 2–3 h/day.

The anthropometric measurements of our study showed that body mass and height mean values were higher in male rowers (182.1 ± 6.6 cm, 75.3 ± 5.3 kg) than female rowers (169.9 ± 6.7 cm, body mass: 61.9 ± 6.0 kg). Results also showed that height and body mass correlate with rowing performance in male and female rowers. Furthermore, height was the best predictor of performance in male rowers (R^2^ = 0.56, *p* < 0.003). Although there is no scientific evidence on studies of comparative analysis of a complete body composition profile between male and female rowers in traditional rowing, some of the rowers’ characteristics in studies on traditional rowing are consistent with this study. Elite traditional male rowers from the Spanish First League of Traineras (ACT) showed a very similar body mass and height to our male rowers (77.0 ± 7.6 kg and 181.1 ± 3.4 cm) [37]. However, other studies have indicated that elite traditional male rowers were heavier (84.4 ± 6.3 kg) but with similar height (182.5 ± 5.2 kg) [8]. In other studies, traditional male rowers of lower competitive level were shorter (178.4 ± 8.9 cm) but with similar body mass (77.3 ± 7.9 kg) [26]. The winners of the Traineras women’s league and the La Concha championship [38] coincide with height (168.2 ± 6.3 cm) and body mass (61.2 ± 4.4 kg) results of our study. However, female rowers in Sebastiá-Amat et al. [26] were slightly shorter (166.3 ± 7.5 kg) and lighter (59.9 ± 8.3 cm). It is generally accepted that height is a very important anthropometric characteristic for rowing performance because a greater height increases the amplitude of the drive in the water [7,39]. The results of the studies on Olympic rowing follow the same trend in both height and body mass. Male Olympic rowers reach heights over 190 cm and weigh more than 90 kg, while female rowers exceed 180 cm in height with a body mass of around 75 kg [14,18,19,40]. These discrepancies may be because the height of rowers can be a differentiating characteristic between higher and lower performance in traditional modalities, while the same does not happen with body mass. However, the rowers of the Trainera boat seem to have a higher average weight than the Llaüt rowers. This may be due to the difference in the number of rowers in each boat and the need for the bow rowers to be lighter, lowering the average weight in the Llaüt for correct navigation. Several studies suggest that traditional rowing boats require rowers with different anthropometric profiles, especially in the bow, due to the hydrodynamics of the boat when competitions are held at sea and the body mass placement of the rowers is important [2,8,38].

In the same way, BMI has reached higher values in male rowers (22.8 ± 1.3 kg/m^2^) than in female rowers (21.4 ± 1.0 kg/m^2^). Studies about male traditional rowers have shown values of BMI greater than 23 kg/m^2^ [34,37] and 24 kg/m^2^ [7,8,26]. However, BMI of our male rowers is similar to lightweight Olympic (22.1 ± 0.3 kg/m^2^) since the rowers in the present study weighed less than the rowers in both traditional and Olympic rowing studies. Finally, BMI values of our female rowers were similar to other traditional rowing (21.7 ± 2.6 kg/m^2^) [26] and Olympic rowing studies (21.6 ± 6.1 kg/m^2^) [19]. In this latest study, Winkert et al. suggested a body composition with high lean body mass and adequate power to body mass ratios instead of a high body mass, because increased body mass and BMI showed a negative effect on career attainment.

The skinfolds and mean of the sum of 8 skinfolds have a larger value in female rowers (88.0 ± 17.6 mm) than in male rowers (58.5 ± 12.4 mm). It is important to know the values obtained from the skinfold measurement, as it is used to predict fat mass. Furthermore, these differences were expected because women have 6 to 11 percent more body fat than men. Studies show that estrogens reduce a woman’s ability to burn energy after eating, thus storing more fat in the body [41]. However, female rowers have lower values in girths and breadths, both in the upper body and in the lower body, except for hip girths with very little difference. In contrast to the scientific literature, it seems that the male rowers in our study have lower values in the sum of skinfolds (67.3 ± 15.6 mm) compared to elite traditional rowers [8]. Compared to rowers participating in the 2000 Sydney Olympic Games [18], the sum of skinfolds of the male rowers in the present study is between open-class (65.3 ± 17.3 mm) and lightweight (44.7 ± 8.1 mm). In the case of female traditional rowers, the values are very similar to the values reached by the open-class female rowers (89.0 ± 23.6 mm). The sum of skinfolds of the lightweight female rowers was only 59.7 ± 12.4 mm.

In our study, male traditional rowers have similar values of muscle mass (46.7 ± 2.0%) compared to other traditional rowing studies of competitions of the same distance as the present study: 46.5 ± 2.0% [34], and large values than other studies of competitions over much longer distances where slimmer rowers are needed.: 43.5 ± 2.0% [42] 43.3 ± 2.4% [8]. According to other studies, female rowers achieved a lower percentage of muscle mass (39.1 ± 2.1%). However, muscle mass for female rowers may be a good predictor of performance in traditional rowing in our study (R^2^ = 0.68, *p* < 0.002) and in the scientific literature [26]. This may be because women have much less testosterone than men and due to the influence of this hormone on the development of strength and muscles, women are less likely to develop equal strength and muscle size than men [43]. Therefore, the difference in strength between women is greater than between men and this characteristic seems to become a differentiating factor in performance. In female rowers. On the other hand, female rowers achieved higher fat mass (15.4 ± 3.1%) than male rowers (10.3 ± 2.1%), according to the description of elite rowers of González [38], where female rowers reached 16.3 ± 5.5% and male rowers 7.8 ± 1.1%. Studies on elite male rowers showed lower percentages of fat mass (9.9 ± 2.0%) [8] than studies conducted with sub-elite rowers (14.2 ± 4.4%) [25]. The percentage ranges for international Olympic rowers was 6% to 10% and 11% to 15% for male and female, respectively [44].

In the only two studies published to date on anthropometric profile of traditional male rowers, endo-mesomorph somatotypes were found (3.5-4.7-2.4 [8] and 3.3-3.9-2.2 [42]). However, the mean somatotype in the present study is categorized as ecto-mesomorph (1.8-4.5-3.0) for male rowers, and balanced mesomorph (2.9-3.1-2.9) for female rowers following Carter and Heath [33] where in ecto-mesomorph somatotype, the mesomorphy component is dominant and the ectomorphy component is greater than the endomorphy component; and in balanced mesomorph somatotype, the mesomorphy component is dominant and the endomorphy and ectomorphy components are equal. Our results coincide with the results of Olympic rowers where male rowers had a somatotype defined as ecto-mesomorph (1.9-5.0-2.5) and female rowers a somatotype categorized as balanced mesomorph (2.8-3.8-2.6). The difference between studies may be due to the competition distances of the rowers analyzed from each study: 14,816 m [42] and 5556 m [8]. On the other hand, the rowers in the present study had to row over 1400 m, a distance much more like the 2000 m that Olympic rowers must cover.

Results of the present study should be interpreted with caution because the main limitation of this study lies in the sample size. Also, some of the results are the product of predictive equations rather than direct measurements. Therefore, they can be used as references but should be interpreted in the context of individual characteristics and needs. In addition, it is important to bear in mind that the evaluations have been individual and on rowing ergometer, while the athletes compete in collective boats that may require different profiles as mentioned above. Future research should analyze the differences by position in the boat: bow, stern, and rest rowers. The need for more heterogeneous rowers in traditional rowing boats compared to Olympic rowing may yield a more detailed profile by position. Furthermore, it would be interesting to determine the relationships between the anthropometric profile and rowing performance in male and female traditional rowers to define which characteristics might be most relevant to each one.

## 5. Conclusions

This study analyzed and compared the anthropometric profile, body composition, and somatotype in male and female traditional rowers, and the role of these variables in the prediction of rowing performance. The results showed that male traditional rowers were significantly taller and heavier, with higher values of girths and breadths, in addition to greater muscle mass. Female traditional rowers reached higher sum of skinfolds and greater fat mass. The mean somatotype for male and female traditional rowers was ecto-mesomorph and balanced mesomorph, respectively, with significant differences in the mesomorph region of male rowers and the endomorph region of female rowers.

Large values of body mass and height correlated with rowing performance in male and female rowers, highlighting height as the best predictor of rowing performance for male traditional rowers. Furthermore, muscle mass positively correlated in female rowers, being the best predictor for rowing performance.

This study shows a detailed anthropometric description of traditional rowers competing at the national level that can be useful as reference values for coaches and rowers. Furthermore, the study shows different variables that can be used to control training and increase rowing performance, such as body and muscle mass, and to identify potential talents in young athletes thanks to characteristics such as height.

## Figures and Tables

**Table 1:** Mean values of anthropometric measurements and difference between male and female rowers.

	Male (*n* = 13)		Female (*n* = 11)		*t*-Test		Cohen’s *d*	
	Mean ± SD	Min–Max	Mean ± SD	Min–Max	*p*	95% CI	*d*	Effect Size
**Basic measurements**								
Age (years)	27.3 ± 5.1	20.0–37.0	27.7 ± 4.3	21.0–35.0	0.831	−4.5–3.6	0.08	Trivial
Body mass (kg)	75.3 ± 5.3 *	66.0–83.1	61.9 ± 6.0	51.8–69.6	<0.001	8.7–18.2	2.4	Very large
Height (cm)	182.1 ± 6.6 *	174.0–193.0	169.9 ± 6.7	160.0–178.0	<0.001	6.5–17.8	1.8	Large
BMI (kg/m^2^)	22.8 ± 1.3 *	20.8–24.5	21.4 ± 1.0	20.1–23.6	0.010	0.4–2.3	1.2	Large
**Skinfolds**								
Triceps (mm)	6.2 ± 1.7	3.0–10.0	12.1 ± 2.4 *	7.0–15.0	<0.001	−7.6–−4.1	2.9	Very large
Biceps (mm)	2.7 ± 0.7	2.0–4.0	4.4 ± 1.4 *	2.0–6.0	0.004	−2.7–−0.6	1.6	Large
Subscapular (mm)	7.7 ± 1.4	6.0–10.0	8.1 ± 1.8	6.0–11.0	0.463	−1.9–0.9	0.3	Small
Iliac crest (mm)	9.7 ± 3.4	5.0–15.0	13.4 ± 3.8 *	9.0–21.0	0.020	−6.7–−0.6	1.0	Moderate
Supraspinal (mm)	6.8 ± 1.9	4.0–10.0	8.5 ± 2.7	6.0–13.0	0.088	−3.7–0.3	0.8	Moderate
Abdominal (mm)	10.2 ± 3.4	6.0–16.0	12.1 ± 4.5	6.0–20.0	0.222	−5.4–1.3	0.5	Small
Front thigh (mm)	10.1 ± 2.9	6.0–15.0	18.4 ± 4.5 *	11.0–24.0	<0.001	−11.4–−5.1	3.5	Very large
Calf (mm)	5.1 ± 1.7	3.0–9.0	10.9 ± 3.6 *	6.0–18.0	<0.001	−8.3–−3.3	2.0	Very large
Σ 8 skinfolds (mm)	58.5 ± 12.4	37.0–75.0	88.0 ± 17.6 *	61.0–117.0	<0.001	−42.1–−16.9	2.0	Very large
**Girths**								
Relaxed arm (cm)	31.0 ± 2.0 *	27.5–34.0	26.5 ± 1.5	24.0–29.0	<0.001	3.0–6.0	2.5	Very large
Tensed arm (cm)	34.6 ± 2.1 *	30.5–37.5	28.6 ± 1.2	27.0–30.8	<0.001	4.5–7.4	3.4	Very large
Thigh (cm)	54.1 ± 2.2 *	48.5–56.5	52.1 ± 2.3	48.0–56.0	0.034	0.2–4.0	0.9	Moderate
Medial calf (cm)	37.2 ± 3.3	27.0–39.5	36.0 ± 1.9	33.0–39.5	0.297	−1.1–3.5	0.4	Small
Waist (cm)	79.6 ± 3.0 *	74.5–85.0	70.5 ± 3.9	65.0–76.0	<0.001	6.2–12.1	2.7	Very large
Hip (cm)	95.3 ± 3.4	88.0–99.0	95.9 ± 4.9	89.5–106.0	0.763	−4.0–3.0	0.1	Trivial
**Breadths**								
Humerus (cm)	7.1 ± 0.3 *	6.5–7.5	6.3 ± 0.3	5.7–6.6	<0.001	0.6–1.1	2.7	Very large
Femur (cm)	9.7 ± 0.4 *	9.0–10.0	9.0 ± 0.4	8.5–9.5	<0.001	0.3–1.0	1.8	Large
Stylion (cm)	5.7 ± 0.2 *	5.4–6.5	5.1 ± 0.2	4.7–5.5	<0.001	0.4–0.9	3.0	Very large

BMI: Body Mass Index; SD: standard deviation; min: minimum; max: maximum; CI: confidence interval; *: statistically significance between male and female rowers (*p* < 0.05).

**Table 2:** Descriptive data and comparative analysis of body composition and somatotype between male and female rowers.

	Male (*n* = 13)		Female (*n* = 11)		*t*-Test		Cohen’s *d*	
	Mean ± SD	Min–Max	Mean ± SD	Min–Max	*p*	95% CI	*d*	Effect Size
**Body composition**								
Fat mass (%)	10.3 ± 2.1	6.6–13.1	15.4 ± 3.1 *	10.7–20.5	<0.001	−7.3–−3.0	2.0	Very large
Muscle mass (%)	46.7 ± 2.0 *	43.1–49.7	39.1 ± 2.1	35.2–43.5	<0.001	5.8–9.3	3.7	Very large
Bone mass (%)	16.2 ± 2.2	10.1–18.6	16.0 ± 0.8	14.7–17.4	0.754	−1.2–1.6	0.1	Trivial
Residual mass (%)	26.4 ± 1.9	22.9–29.0	29.4 ± 1.9 *	26.2–32.5	<0.001	−4.6–−1.4	1.6	Large
Fat mass (kg)	7.8 ± 1.9	4.38–10.4	9.6 ± 2.4	5.7–12.9	0.051	−3.6–0.1	0.8	Moderate
Muscle mass (kg)	35.1 ± 2.3 *	31.9–38.6	24.2 ± 2.4	20.4–28.0	<0.001	8.9–12.9	4.7	Very large
Bone mass (kg)	12.5 ± 1.2 *	11.1–15.4	9.9 ± 1.0	8.4–11.2	<0.001	1.7–3.5	2.3	Very large
Residual mass (kg)	19.9 ± 1.9 *	16.7–22.6	18.2 ± 1.9	14.7–20.1	0.039	0.1–3.3	0.9	Moderate
**Somatotype**								
Endomorphy	1.8 ± 0.5	1.0–2.4	2.9 ± 0.6 *	2.1–4.0	<0.001	−1.5–−0.6	2.0	Very large
Mesomorphy	4.5 ± 0.9 *	3.1–6.5	3.0 ± 0.7	1.7–4.0	<0.001	0.9–2.2	1.8	Large
Ectomorphy	3.0 ± 0.8	1.8–3.9	2.9 ± 0.6	1.5–3.5	0.685	−0.5–0.7	0.1	Trivial
SAM	1.1 ± 0.5	0.5–2.3	0.9 ± 0.5	0.5–2.0	0.242	−0.7–0.2	0.4	Small

BMI: Body Mass Index; SD: standard deviation; min: minimum; max: maximum; CI: confidence interval; *: statistically significance between male and female rowers (*p* < 0.05).

**Table 3:** Stepwise multiple regression model of rowing performance.

Rowers	Equation	R^2^	Adj. R^2^	SEE	*p*
Male	W_2000m_ = 2.23 × Height (cm) − 140.31	0.56	0.52	13.72	0.003
Female	W_2000m_ = 7.18 × Muscle mass (kg) − 4.18	0.68	0.65	12.28	0.002

SEE: standard error of estimate; W: power.

## Data Availability

The data presented in this study are available on reasonable request from the corresponding author.
